# Regional gender differences in an autosomal disease result in corresponding diversity differences

**DOI:** 10.1038/s41598-019-41905-8

**Published:** 2019-04-02

**Authors:** Shenmin Guan, Yingying Zhao, Xiao Zhuo, Wenhui Song, Xiaorui Geng, Huanming Yang, Jian Wang, Xinhua Wu, Jinlong Yang, Xin Song, Le Cheng

**Affiliations:** 1 Shenzhen University Health Sciences Center, School of Basic Medical Sciences, Department of Physiology, Shenzhen, 518061 China; 2 BGI-Yunnan, BGI-Shenzhen, Kunming, 650106 China; 3 Dali University First affiliated Hospital, Dali, 671000 China; 4 The Third Affiliated Hospital of Kunming Medical University (Tumor Hospital of Yunnan Province), Kunming, 650000 China; 5 Shenzhen Longgang ENT Institute, Shenzhen, 518100 China; 6 Puer University, Puer School of BGI-Yunnan, Puer, 665000 China; 7 Dali University, School of Basic Medical Sciences, Dali, 671003 China; 8 BGI-Shenzhen, Shenzhen, 518083 China; 9 James D. Watson Institute of Genome Sciences, Hangzhou, 310058 China

## Abstract

Regional gender differences in autosomal chromosome disorders have been observed repeatedly. However, the corresponding diversity changes remain unconfirmed. By analyzing previously published thalassemia data from the Dai people in Dehong and Xishuangbanna (two regions in Yunnan Province, China), we found that several sequence types, including HBA CNV and HBB mutations, significantly depend on gender in Xishuangbanna but not in Dehong. With the supportive evidence from previous researches, we accept that some certain mutations depend on gender regionally. This association seems peculiar. It is among one common people on a small geographical scale, while other recorded thalassemia gender difference varies by ethnics and continent.

## Introduction

In past decades, various gender differences in autosomal chromosome disorders have been observed^1–6^, and corresponding differences in related mutation frequencies were found^7,8^. However, the regionality of these genetic gender dependencies has never been investigated, even though some gender differences in autosomal disorders are regional^9–15^. Thus, we aimed to determine the regional gender dependency of genetic factors.

We focused on thalassemia among the Dai people in Dehong and Xishuangbanna (two regions in Yunnan Province, China).

Thalassemia is an autosomal chromosome disorder^16^ (https://en.wikipedia.org/wiki/Thalassemia) with various gender differences^4–6,9–15^. The severity of the bone disease and cardiac disorder associated with thalassemia varies by gender^4–6^. A significant association between gender and alloimmunization during transfusion was found in African Americans^9^ but not in Indians^10,11^. Similar phenomena were found for selection due to malaria. In Kenya, malaria risk is higher for females than for males, and in India, this pattern is reversed^12,13^. However, females in India are more likely to die from severe malaria, even if differences in therapy between genders are eliminated; this likelihood is not observed in Ethiopia^14,15^. The cases above involve gender-dependent fitness, potential mortality and regional selection. These effects could result in a regional diversity difference between genders.

Dehong and Xishuangbanna are located in the southern part of Yunnan Province, China. Both locations have a hot climate year round, with rainy weather during the wet season. The inhabitants are at high risk of malaria^17,18^ and have a high incidence of thalassemia^19–21^. According to studies performed in the 1980s and 2010s, abnormal hemoglobin was found to be frequent in various minorities^21,22^. Additionally, a genetic test in 2011 revealed that the carrier rates for pathogenic thalassemia mutations among minority children were 46% in Dehong and 40% in Xishuangbanna^23^. This result was supported by another genetic test in 2013^24^.

The Dai people are populous in Dehong and Xishuangbanna and have an extraordinarily high frequency of pathogenic thalassemia mutations^25,26^. Although the two regions are located close to each other and have almost the same ethnic composition, neglected regional gender differences were found when we interpreted Yao’s work^27^ (see the supplement for our interpretation of previous research). The beta thalassemia incidence among Dai children (by a hemoglobin test) depends significantly on gender in three regions of Xishuangbanna, but not in any observed region of Dehong.

Our work is based on a previous study that was published in 2017^28^. In this study, 951 Dai individuals from Dehong and Xishuangbanna were tested with next-generation DNA sequencing, and basic personal information was recorded (see DataForAna in S1_Table.xls). We mined the data and compared the neglected association factors of sequence type frequency in two regions. If the frequencies are related to gender, we will accept the hypothesis that thalassemia gender differences result in consequent diversity differences between genders. If some of these relationships are significant in only one region, we will conclude that the gender-dependent diversity difference is regional.

## Results

**Figure 1:**
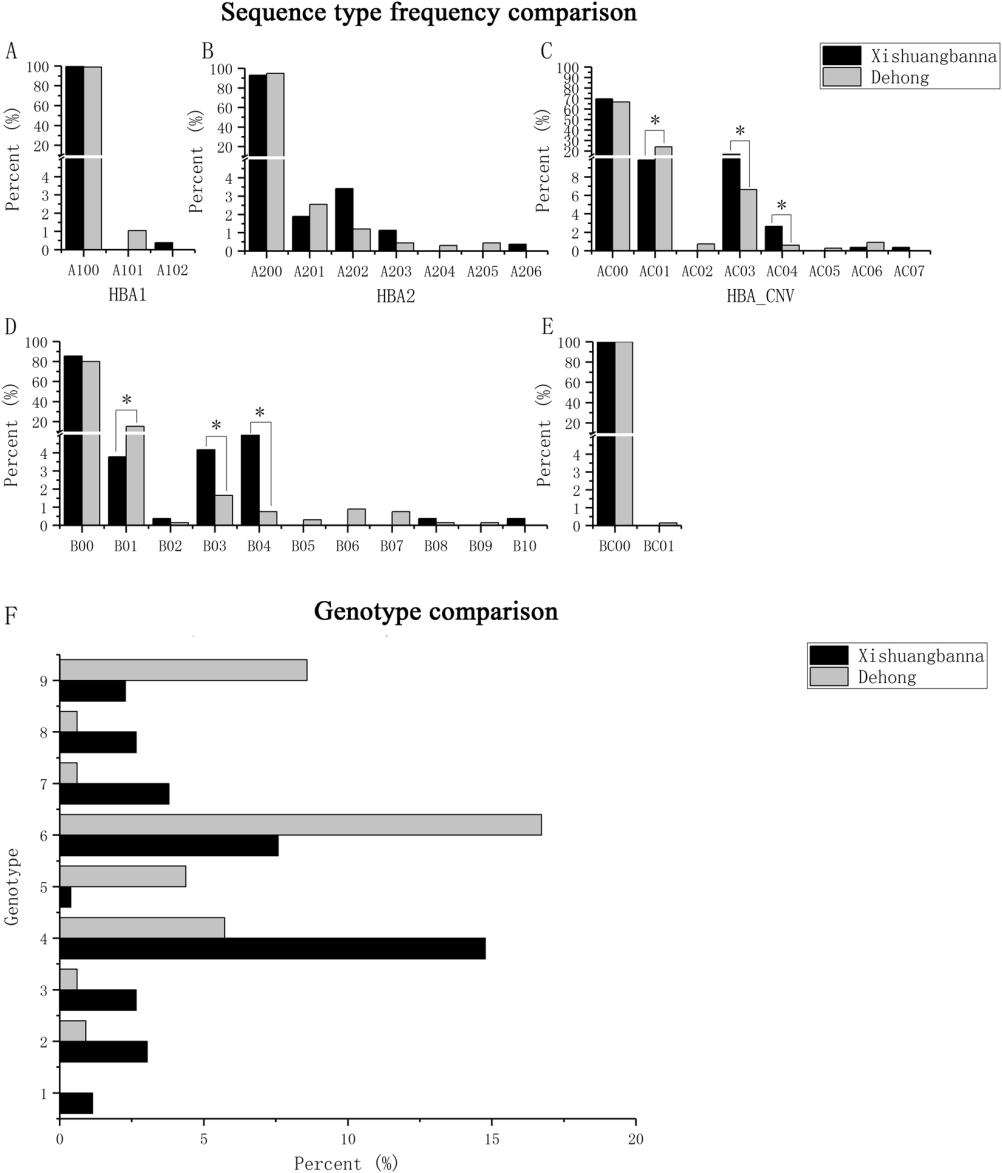
Diversity comparison. In panels A-E, the frequency of each sequence type is presented. “*” indicates that the difference is significant. Panel F shows that all the genotypes differed significantly between the two regions. The paired code name and type annotation listed in the legend for each genotype was used in the rest of this article, including in the description of genotypes and the identification of genotypes for association rules. (**A**) HBA1: hemoglobin, alpha 1. A100 = normal, A101 = c.95 + 1G > A, A102 = c.98T > A. (**B**) HBA2: hemoglobin, alpha 2. A200 = normal, A201 = c.427T > C (het), A202 = c.369C > G (het), A203 = c.369C > G (hom), A204 = c.1delA (hom), A205 = c.427T > C (hom), A206 = c.377T > C (hom). (**C**) HBA_CNV: hemoglobin, alpha, copy number variation. AC00 = normal, AC01 = αα/−α^3.7^, AC02 = αα/ −α^3.7^(α1 and α2 fusion after deletion in another chain), AC03 = αα/–^SEA^, AC04 = αα/−α^4.2^, AC05 = −α^3.7^/= ^SEA^, AC06 = −α^3.7^/−α^3.7^, AC07 = αα/ααα. (**D**) HBB: hemoglobin, beta. B00 = normal, B01 = c.79G > A (het), B02 = c.217dupA (het), B03 = c.126_129delCTTT (het), B04 = c.52A > T (het), B05 = c.316-238C > T (het), B06 = c.79G > A (hom), B07 = c.380T > G (het), B08 = c.410G > A (het), B09 = c.126delC (het), B10 = c.92 + 1G > T (het). (**E**) HBB_CNV: hemoglobin, beta, copy number variation. BC00 = normal, BC01 = 1-HBB. Here, “het” and “hom” denote heterozygote and homozygote. The details of the code names can be seen in S2_Table.xls. (**F**) Each genotype is a combination of sequence types, and every combination is described with the involved sequence types. Genotype 1 = A100, A203, AC03, B00, BC00; Genotype 2 = A100, A202, AC00, B00, BC00; Genotype 3 = A100, A200, AC04, B00, BC00; Genotype 4 = A100, A200, AC03, B00, BC00; Genotype 5 = A100, A200, AC01, B01, BC00; Genotype 6 = A100, A200, AC01, B00, BC00; Genotype 7 = A100, A200, AC00, B04, BC00; Genotype 8 = A100, A200, AC00, B03, BC00; Genotype 9 = A100, A200, AC00, B01, BC00.

The frequency of each sequence type is shown in Fig. 1 (Fig. 1A–E, see CompareTypeFrequency01 in S1_Table.xls). A total of 31 sequence types were recorded. Most sequence types had a frequency of less than 5%, and several sequence types appeared to differ between Xishuangbanna and Dehong. By Fisher’s exact test, the differences in six sequences types were found to be significant; these sequence types include three hemoglobin alpha copy number variations (“αα/−α^3.7^”, “αα/−−^SEA^”, and “αα/−α^4.2^”) and three hemoglobin beta mutations (“HBB: c.79G > A(het)”, “HBB: c.126_129delCTTT(het)”, and “HBB: c.52A > T(het)”) (Fig. 1A–E). Although the sequence type number suggested that the number of combinations of these sequence types would be large (Fig. 1A–E, see CompareTypeFrequency01 in S1_Table.xls), only 53 genotypes were observed. The frequencies of nine genotypes differed significantly between the two regions and are shown in Fig. 1 (Fig. 1F, see CompareTypeFrequency02 in S1_Table.xls).

**Figure 2:**
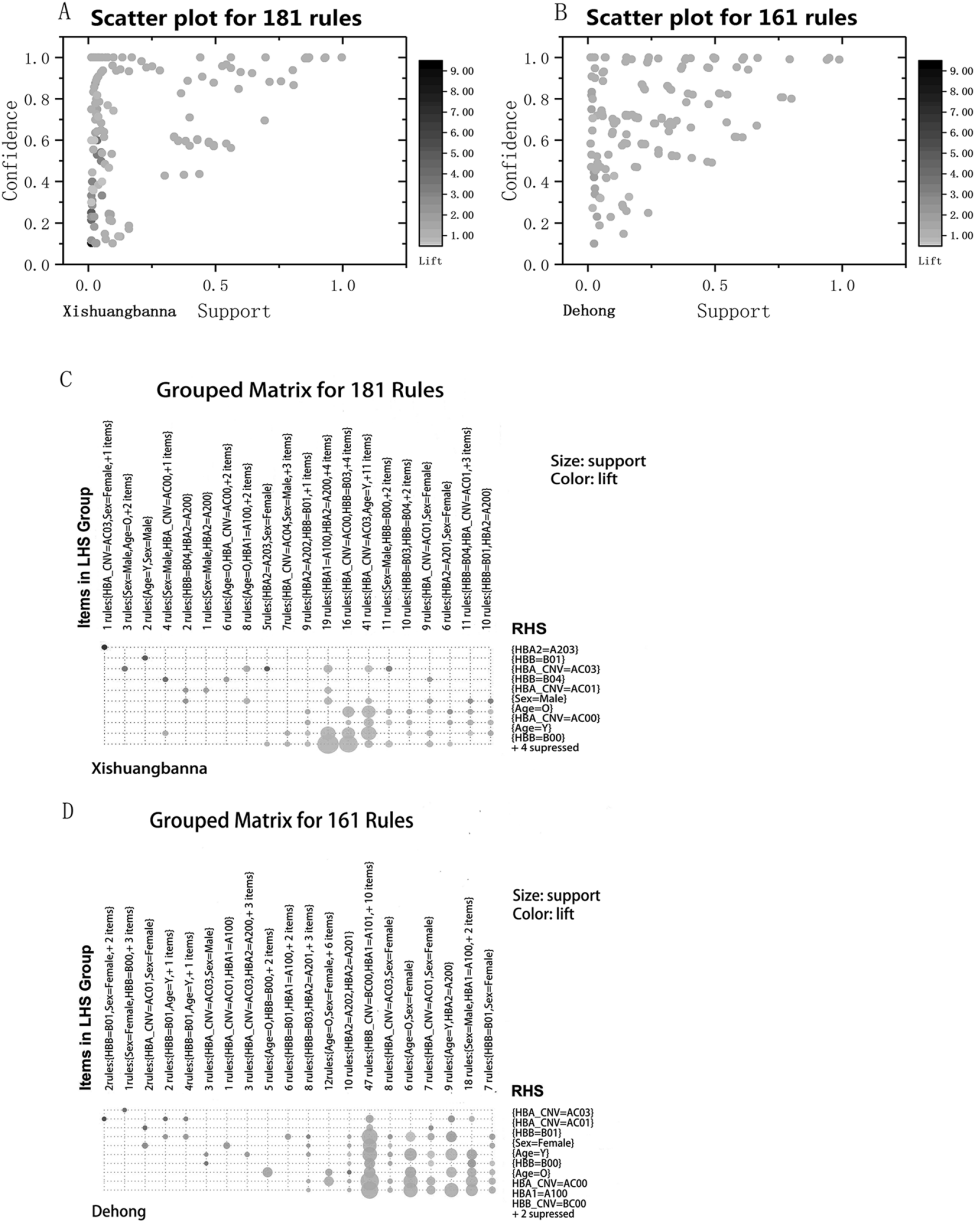
Scatter diagram and classification diagram for association rules. The support, evidence and lift in the scatter diagrams were given by the apriori algorithm and are the most important properties of an association rule. The high lift indicates that the identified rule cannot be caused by randomness. In the classification diagrams, the association rules were classified by the left-hand side (LHS) of the implication. Rules that share more common property-value pairs in the LHS are thought to be more similar.

Discounting redundant rules, 181 and 161 association rules were found in Xishuangbanna and in Dehong, respectively (see AssociationRulesBN, AssociationRulesDH in S1_Table.xls). In both regions, the rules with the highest lift all had support values less than 0.1, and in most other cases, the lift was near 1 (Fig. 2, see AssociationRulesBN, AssociationRulesDH in S1_Table.xls). In each region, the rules are divided into 20 groups by the left-hand side (LHS) of the implication (Fig. 2). The rule classification shows that the rules identified by the apriori algorithm are regional in a sense. In Xishuangbanna, the rules with the highest lift always have an LHS containing “HBA_CNV = AC03, Sex = Female” or “Sex = Male, Age = O” (AC03 is for “αα/ −− ^SEA^”). In Dehong, the lifts are highest when the LHS contains “HBB = B01” and “sex = Female” (B01 is for “HBB: c.79G > A(het))”.

**Figure 3:**
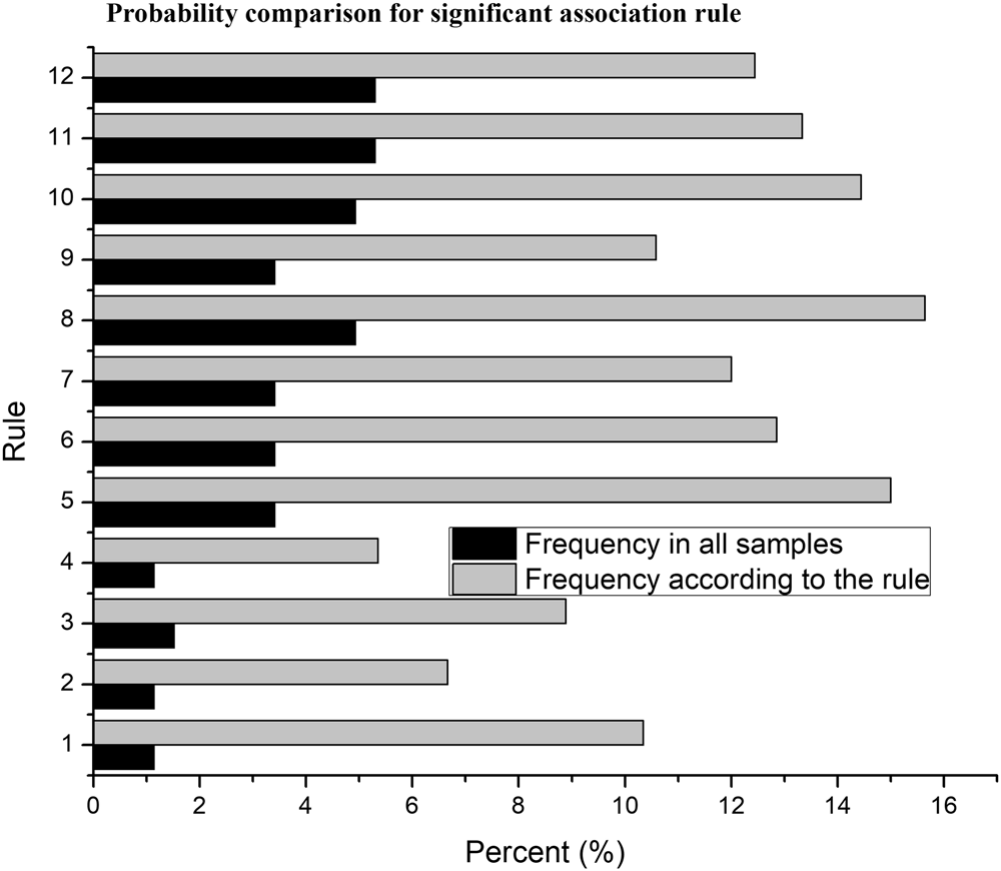
Probability comparison for significant association rules. Each pair of bars represents a significant rule. This figure shows the difference between the joint probability and the conditional probability corresponding to each significant association rule. The joint probability is described by the percentage of the samples corresponding to the right-hand side (RHS) of the rule, which indicates the frequency in all samples. The conditional probability is described by the percentage of the samples corresponding to both the LHS and the RHS (left- and right-hand sides of the rule), which indicates the frequency according to the rule. Rule 1 {Sex = Female, HBA_CNV = AC03, HBB = B00} => {HBA2 = A203}, Rule 2 {HBA2 = A203} => {HBA_CNV = AC03}, Rule 3 {Sex = Male, Age = Y} => {HBB = B01}, Rule 4 {Sex = Male, HBA2 = A200, HBA_CNV = AC00} => {HBB = B04}, Rule 5 {Sex = Male, Age = O, HBA2 = A200, HBB = B00} => {HBA_CNV = AC03}, Rule 6 {Sex = Male, Age = O, HBB = B00} => {HBA_CNV = AC03}, Rule 7 {Sex = Male, Age = O, HBA2 = A200} => {HBA_CNV = AC03}, Rule 8 {Sex = Male, HBA2 = A200, HBB = B00} => {HBA_CNV = AC03}, Rule 9 {Sex = Male, Age = O} => {HBA_CNV = AC03}, Rule 10 {Sex = Male, HBB = B00} => {HBA_CNV = AC03}, Rule 11 {HBA2 = A200, HBA_CNV = AC03} => {Sex = Male}, Rule 12 {Sex = Male} => {HBA_CNV = AC03}. The significant association rules found in Xishuangbanna were divided into three groups here.

Most of the associations were not accepted (see Method). According to the Fisher’s exact test (see FTestAssociationRulesBN, FTestAssociationRulesDH in S1_Table.xls), the odds ratios (see Method) for 12 rules from Xishuangbanna departed from 1 significantly, and these rules were thought to be acceptable (Fig. 3, see FTestAssociationRulesBN in S1_Table.xls). In contrast, no rules from Dehong had an odds ratio that significantly departed from 1, although two rules had p values of 0.0504 and 0.07 (see FTestAssociationRulesDH in S1_Table.xls). These p values could be due to the rarity of the relevant sequence types, and these rules might be judged to be significant in future studies with a much larger sample size, but they were not considered significant here.

The first group relates to the combination of certain pathogenic mutation types. Only one rule is in this group, “{HBA2 = A203} => {HBA_CNV = AC03}”. This rule holds that a person with the mutation “HBA2: c.369C > G (hom)” (code with “A203”) is more likely to carry “αα/−−^SEA^” (coded with “AC03”).

The second group suggests an association between being female and having certain sequence type combinations. This group also contains only one rule, “{Sex = Female, HBA_CNV = AC03, HBB = B00} => {HBA2 = A203}”. This rule holds that with the pathogenic mutation “αα/−−^SEA^” (coded with “AC03”) and normal HBB, a female is more likely to carry “HBA2: c.369C > G (hom)” (coded with “A203”).

**Figure 4:**
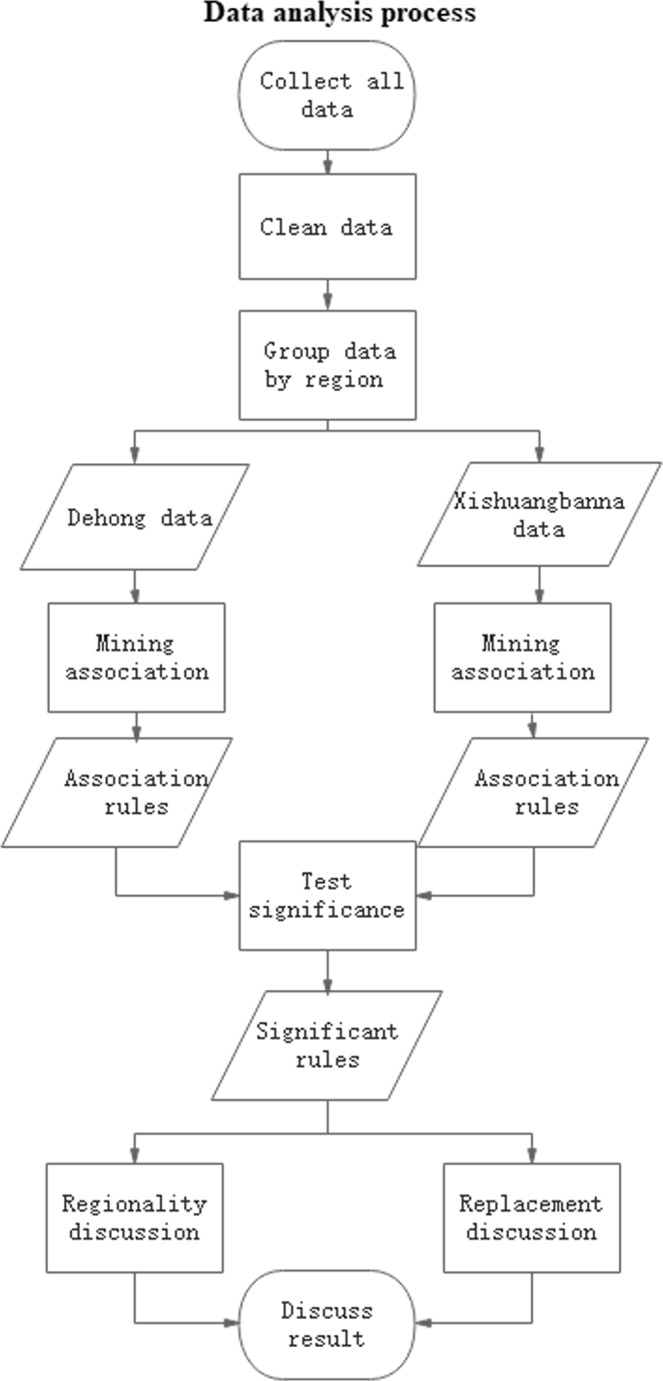
Data analysis process. The process includes multiple steps: data cleaning, association mining, association rule significance testing, association regionality discussion, and association rule replacement discussion. The data were cleaned mainly by removing the items without needed information. The associations were mined with the apriori algorithm. The association rules identified by apriori were tested with the Fisher’s exact test. Then, the regionality and replacement of the significant rules were discussed. When discussing regionality, each significant rule found in a region was tested in the other region. If a rule was significant in one region but not in the other, it was thought to be regional. When discussing the rationality of replacing an apriori rule with a simpler rule, a comparison was made between two conditional probabilities, one for the apriori rule and the other for a given simpler rule that could replace the former rule. If the probabilities differed from each other significantly, the replacement was thought not to be rational. At last, a permutation test was designed for the Xishuangbanna rules including “Sex” in discussion.

The third group indicates an association among male sex, age and certain combinations of sequence types. This group has nine rules, all with “Sex = Male” (Fig. 3, rules 3–11). This group can be summarized with a few knowledge points. First, the frequency of “HBB: c.79G > A(het)” (coded with “B01”) is higher among young men (see “{Sex = Male, Age = Y} => {HBB = B01}”). Second, when both HBA2 and HBA CNV are normal, a higher frequency of “HBB: c.52A > T(het)” (coded with “B04”) can be expected in men (see “{Sex = Male, HBA2 = A200, HBA_CNV = AC00} => {HBB = B04}”). The rest of the rules (Fig. 4, rules 5–12) in this group can be summed as the possibility that “αα/−−^SEA^” varies among men according to HBA2, HBB and age.

The rules that were significant in Xishuangbanna (Fig. 3) were tested in Dehong. The results of these additional Fisher’s exact tests suggested that no rule is significant in both region (see RuleBNInDH in S1_Table.xls), since most p values were near 1 in Dehong. It was accepted that the significant rules found in Xishuangbanna are regional.

A conditional probability comparison was used to discuss whether a given simpler rule can serve as a replacement for a rule produced by the apriori function (see ComparisonBN in S1_Table.xls). The Fisher’s exact test outputted the p values for 21 comparisons that were less than or equal to 0.05 (Table 1.). This outcome means that these rules identified by apriori cannot be replaced with the given simpler rules. However, other replacements (see ComparisonBN in S1_Table.xls) may be rational.

**Table 1:** Rule comparison.

No	Rule	Simpler Rule	P Value
1	{Sex = Female, HBA_CNV = AC03, HBB = B00} => {HBA2 = A203}	{Sex = Female} => {HBA2 = A203}	0.0252
2	{Sex = Female, HBA_CNV = AC03, HBB = B00} => {HBA2 = A203}	{HBB = B00} => {HBA2 = A203}	0.026
3	{Sex = Female, HBA_CNV = AC03, HBB = B00} => {HBA2 = A203}	{Sex = Female, HBB = B00} => {HBA2 = A203}	0.0346
4	{Sex = Male, Age = Y} => {HBB = B01}	{Age = Y} => {HBB = B01}	0.0332
5	{Sex = Male, HBA2 = A200, HBA_CNV = AC00} => {HBB = B04}	{HBA2 = A200} => {HBB = B04}	0.0484
6	{Sex = Male, Age = O, HBA2 = A200, HBB = B00} => {HBA_CNV = AC03}	{Age = O} => {HBA_CNV = AC03}	0.0164
7	{Sex = Male, Age = O, HBA2 = A200, HBB = B00} => {HBA_CNV = AC03}	{HBA2 = A200} => {HBA_CNV = AC03}	0.0028
8	{Sex = Male, Age = O, HBA2 = A200, HBB = B00} => {HBA_CNV = AC03}	{HBB = B00} => {HBA_CNV = AC03}	0.0045
9	{Sex = Male, Age = O, HBA2 = A200, HBB = B00} => {HBA_CNV = AC03}	{Age = O, HBA2 = A200} => {HBA_CNV = AC03}	0.0177
10	{Sex = Male, Age = O, HBA2 = A200, HBB = B00} => {HBA_CNV = AC03}	{Age = O, HBB = B00} => {HBA_CNV = AC03}	0.0251
11	{Sex = Male, Age = O, HBA2 = A200, HBB = B00} => {HBA_CNV = AC03}	{HBA2 = A200, HBB = B00} => {HBA_CNV = AC03}	0.0044
12	{Sex = Male, Age = O, HBA2 = A200, HBB = B00} => {HBA_CNV = AC03}	{Age = O, HBA2 = A200, HBB = B00} => {HBA_CNV = AC03}	0.0442
13	{Sex = Male, Age = O, HBB = B00} => {HBA_CNV = AC03}	{Age = O} => {HBA_CNV = AC03}	0.0236
14	{Sex = Male, Age = O, HBB = B00} => {HBA_CNV = AC03}	{HBB = B00} => {HBA_CNV = AC03}	0.0089
15	{Sex = Male, Age = O, HBA2 = A200} =>{HBA_CNV = AC03}	{Age = O} => {HBA_CNV = AC03}	0.0291
16	{Sex = Male, Age = O, HBA2 = A200} => {HBA_CNV = AC03}	{HBA2 = A200} => {HBA_CNV = AC03}	0.0077
17	{Sex = Male, HBA2 = A200, HBB = B00} => {HBA_CNV = AC03}	{HBA2 = A200} => {HBA_CNV = AC03}	0.0041
18	{Sex = Male, HBA2 = A200, HBB = B00} => {HBA_CNV = AC03}	{HBB = B00} => {HBA_CNV = AC03}	0.0101
19	{Sex = Male, HBA2 = A200, HBB = B00} => {HBA_CNV = AC03}	{HBA2 = A200, HBB = B00} => {HBA_CNV = AC03}	0.0098
20	{Sex = Male, HBB = B00} => {HBA_CNV = AC03}	{HBB = B00} => {HBA_CNV = AC03}	0.0126
21	{HBA2 = A200, HBA_CNV = AC03} => {Sex = Male}	{HBA2 = A200} => {Sex = Male}	0.0183

Because “Sex” was thought to be essential in Xishuangbanna, a permutation test was applied to the significant rules with “Sex” (see Method). The most concerned three rules are {Sex = Male} => {HBA_CNV = AC03}, {Sex = Male, Age = O} => {HBA_CNV = AC03}, and {Sex = Male, Age = Y} => {HBB = B01}. After shuffling “Sex”, the proportions of their Fisher test p values less than 0.01 were 9e-04, 4e-04, 1e-03, respectively; the proportion of p values less than 0.03 were 0.0011 0.0019 0.0066, respectively; the proportion of p values less than 0.05 were 0.0019 0.0067 0.0129, respectively (see PermutationTestBN in S1_Table.xls). These proportions suggest that, “Sex” certainly relates to “AC03” and “B01”.

Based on the 21 comparisons (Table 1), gender and “αα/−−^SEA^” (coded with “AC03”) seem essential. When removing any one of these parameters from the LHS of an apriori rule, the p value for the comparison is less than 0.05.

## Discussion

Although 12 BN rules were listed in Fig. 3, only three rules, {Sex = Male} => {HBA_CNV = AC03}, {Sex = Male, Age = O} => {HBA_CNV = AC03}, and {Sex = Male, Age = Y} => {HBB = B01}, were seriously discussed here, that because they are more credible. They do not include rare mutations which are not able to be well researched with a small data set. Additionally, these rules had been confirmed by permutation test with shuffling “Sex”.

According to these three rules, the frequencies of certain thalassemia mutations depend on gender in Xishuangbanna but not in Dehong. This dependence had never been reported in Dai people from Xishuangbanna, because no genetic data set had been got from them before 2016. However, the three rules are consistent with previous researches^27,29^.

The rules {Sex = Male} => {HBA_CNV = AC03}, {Sex = Male, Age = O} => {HBA_CNV = AC03} are supported by malaria incidence record^29^. These two rules mean that male, especially elder male have more chance to carry mutation “αα/−−^SEA^”, which was selected by malaria in China^30^. The malaria record is from 1981 to 2010^29^. It can be briefly described as the following points:

- In Xishuangbanna, the cumulative malaria incidence from 1981 to 2005 was about 7.5%, that is considerable.
- In Xishuangbanna, only very few malaria incidences were found after 2006.
- In Xishuangbanna, men in the age between 15 and 35 are with the highest malaria risk. Thus, the men’s cumulative incidence from 1981 to 2005 should be greater than 7.5%, which is based on all people, including men and women.

In our research, elder men were (defined as) born since mid 1970s to 1989 (elder than 24 in 2013); younger men were born after 1990. According to their birth year, it is reasonable to accept that, the elder men had been affected much more than younger.

The elder group were older than 15 years at year 2006. When the malaria incidence was considerable (a), they were at the age of highest risk (c). In this group, the men’s cumulative incidence from 1981 to 2005 should be considerable (c). Even so, the incidence might be underestimated. Because the elder men born in mid 1970s did not count the incidence rate, the incidences were not recorded by medical workers at that time.

Comparatively, the younger had much less infection opportunities: before 2005, they were under 15 years old, the record showed that the children of this age were at much lower risk (c); and after 2006, medical prevention made incidences very rare (b) when they were old enough to get high risk.

By the interpretation above, we found a perfect correspondence: more frequent antimalaria mutation “αα/––^SEA^” are among male, especially elder male (according to our rules), exactly the people at highest risk of infection (according to the previous record). This correspondence suggests that, malaria caused selection can make the observed association.

The rule, {Sex = Male, Age = Y} => {HBB = B01}, is consistent with the a detailed anemia investigation^27^. In this investigation, it was showed that beta thalassemia depends on gender among Dai children in Xishuangbanna, while not in Dehong^27^. Considering ‘B01’ is one of the most frequent beta thalassemia mutation type, we conclude that {Sex = Male, Age = Y} => {HBB = B01} might be a cause of the previously observed gender dependence. Moreover, our rule and previously finding on gender dependence^27^ have common regionality: it is not found in Dehong. The cause of this regionality still remains unknown. The exploration of the cause requires more researches on the mechanism.

Considering the consistence with previous researches, we accept that, certain thalassemia mutations depend on gender regionally. Additionally, it is noteworthy that our identified association rule is also a regional gender difference among one native people on a small geographical scale (Dehong and Xishuangbanna are approximately 400 km from each other). In contrast, most thalassemia gender differences vary by continent or ethnicity^7–11^. We believe that this abnormal regionality results from other neglected factors, such as social life, isolation or unknown selection mechanism. Moreover, the regional gender dependence indicates an interesting question: the likelihood of passing an autosomal genetic disorder to offspring might rely on gender regionally. This likelihood proposed by us is hypothetical and also remains unexplored.

Because our study is based on the first genetic data set for Dai people in Xishuangbanna and Dehong, it is the first data mining, an exploratory work. Considering only 35 males and more than 200 females in the Xishuangbanna set, we tried our best to avoid biased conclusion: only the frequent mutations were considered; the conclusions were drawn on the consideration of the supportive evidences from previous researches, of which sample size ranged from hundreds to thousands^27,29^, much bigger than ours. Actually, our conclusion is not based on a single small sample size. The impact by the imperfections of the data set was eliminated as possible. Of course, better data set is necessary for further work.

## Methods

Before analysis, less than twenty samples were removed because of the absence of basic personal information or genetic test results that were needed for subsequent data mining. Ultimately, 264 samples from Xishuangbanna and 664 samples from Dehong were in the analyzed set (see DataForAna in S1_Table.xls).

The first analysis is a comparison study. The frequency of each sequence type (including copy number variation) was compared between two regions. The frequencies of the observed combinations of these types were also compared. Significance was tested with Fisher’s exact test.

The analysis process for association factors is shown in Fig. 4.

The most important analysis is the association between gender and mutation. Only two genes were recorded in the studied data set. In similar contexts, each mutation is usually studied individually^31^. However, when too many mutation types are involved, it is difficult to list all possible associations manually. Thus, association mining was used to generate the hypothesis here. The importance of each outputted association was evaluated by significance tests and previous studies, as in research methods that focus on a few genes^31^, because Bonferroni adjustment cannot apply to such associations. The occurrence of these mutation types is not independent. In most cases, the occurrence of a mutation means that other mutation types are not found in the same gene.

The function ‘apriori’ in the R package ‘aRules’ was used to search for possible associations. Before running the function apriori, the age of each person was converted to a factor that can be considered by the apriori algorithm. Ages greater than the median age were considered old (labeled ‘O’), and ages less than or equal to the median were considered young (labeled ‘Y’). Thereafter, if an association rule involved age, the corresponding logical implication was indicated with an ‘O’ or ‘Y’. Counting the pathogenic mutation types with very low frequency, the parameters of the function apriori were set manually. The parameter ‘support’ was set to 0.01, and the parameter ‘confidence’ was set to 0.1. After searching for possible rules, redundant rules were removed (see the supplement for the R code used in association mining).

We used Fisher’s exact test to determine whether to accept these rules, but the Bonferroni adjustment was not adopted. The reason for this choice was discussed above. A contingency table was designed for the test: N1 was the sample number corresponding to the LHS of the implication of an association rule; N2 was the number of samples fitting the description of both the LHS and RHS (right-hand side) of the implication; N3 was the total number of samples from one region; and N4 was the sample number corresponding to the RHS of the implication. Then, the odds ratio was (N2/N1)/(N4/N3), just the ‘lift’ provided by the function apriori.

In addition, we must note two major considerations. First, certain rules could be identified in Xishuangbanna and not in Dehong. Common support in the function apriori was used in the two regions, but the samples from Xishuangbanna are considerably less than those from Dehong. Second, the rules identified by apriori could be replaced by another simpler rule. The simpler rule shared a common RHS with the apriori rule, while its LHS was comparatively simpler. The rationality of such replacements should be confirmed.

The first consideration, whether the significant rules counted equally in both regions, was addressed with additional tests. When a rule was found to be significant in Xishuangbanna, another Fisher’s exact test was performed in Dehong. If a rule was significant in both regions but not in Dehong, we concluded that the sample size difference has a negligible effect on our analysis and that the regionality of the rules was not misjudged.

The second consideration, replacement of the rule identified by apriori, was addressed with the conditional probabilities based on the simpler rule and the apriori rule.

The possible simpler rules were exhausted with set operations. The LHS of the implication for an identified rule is seen as a set, and each element is a property-value pair (“Property” is the column name of the data set). Thereafter, all possible simpler rules can be listed by enumerating all the nonempty and proper subsets of the LHS of the identified rules.

The rationality of the replacement of an apriori rule with a simpler rule was determined with the ratio between two conditional probabilities. The first conditional probability was for the apriori rule, defined as N2/N1. N1 was the sample number corresponding to the LHS from this rule, and N2 was the sample number corresponding to both the LHS and RHS. The second conditional probability was for the simpler rule, defined as N4/N3. Similarly, N3 and N4 were the sample numbers corresponding to the LHS and to both the LHS and RHS of the simpler rule, respectively. Naturally, the ratio between the two probabilities was computed with (N2/N1)/(/N4/N3). This ratio is equivalent to the odds ratio generated by the Fisher’s exact test. When the ratio departs from 1 significantly, the simpler rule is thought to be distinct from the identified rule, and the replacement is not rational.

After the analysis above, the association rules in Xishuangbanna and property “Sex” was thought to be essential. Thus, an additional permutation test was conducted. In the Xishuangbanna data, the “Sex” label was shuffled for 10000 times, the Fisher exact test was applied to the rules including “Sex”. For each rule, the proportion of p value less than 0.01, 0.03 and 0.05 were recorded.

## Supplementary information

Supplementary file

Dataset3

Dataset 1

Dataset 2
