# Supplementary material for: Regional gender differences in an autosomal disease result in corresponding diversity differences
Source: Sci Rep. 2019 Apr 2;9:5472. doi: 10.1038/s41598-019-41905-8 (PMC6445288; doi:10.1038/s41598-019-41905-8)
Supplement: Supplementary file 1 — Supplementary file [file 41598_2019_41905_MOESM1_ESM.doc]

**Regional gender differences in an autosomal disease result in corresponding diversity differences**

Shenmin Guan^1,2*^, Yingying Zhao^1*^, Xiao Zhuo^2^, Wenhui Song^2^, Xiaorui Geng^5^, Huanming Yang^8,9^, Jian Wang^8,9^, Xinhua Wu^3^, Jinlong Yang^2,6^, Xin Song^4,10^, Le Cheng^2,5,6,7^

1 Shenzhen University, School of Medicine, Department of Physiology, Shenzhen, 518061, China

2 BGI-Yunnan, BGI-Shenzhen, Kunming, 650106, China

3 Dali University First affiliated Hospital, Dali 671000, China

4 The Third Affiliated Hospital of Kunming Medical University (Tumor Hospital of Yunnan Province), Kunming, 650000, China

5 Shenzhen Longgang ENT Institute, Shenzhen, 518100, China

6 Puer University, Puer School of BGI-Yunnan, Puer, 665000, China

7 Dali University, School of Basic Medical Sciences, Dali, 671003, China

8 BGI-Shenzhen, Shenzhen, 518083, China

9 James D. Watson Institute of Genome Sciences, Hangzhou, 310058, China

10 Deceased

*These authors contributed equally to this work.

Shenmin Guan, [blacktulip-g@163.com](mailto:blacktulip-g@163.com)

Yingying Zhao, [zhaoyingying@szu.edu.cn](mailto:zhaoyingying@szu.edu.cn)

Xiao Zhuo, [zhuoxiao@genomics.cn](mailto:zhuoxiao@genomics.cn)

Wenhui Song, [songwenhui_1982@1](mailto:songwenhui@genomics.cn)63.com

Xiaorui Geng, [gengxr0716@163.com](mailto:gengxr0716@163.com)

Huanming Yang, [yanghuanming@genomics.cn](mailto:yanghuanming@genomics.cn)

Jian Wang, [wangjian@genomics.cn](mailto:wangjian@genomics.cn)

Xinhua Wu [, 13987286915@139.com](mailto:(13987286915@139.com))

Correspondence: Le Cheng [(chengle@genomics.cn),](mailto:(chengle@genomics.cn),) and Xin [Song(songxin68@126.com),](mailto:Song(songxin68@126.com),) Jinlong Yang [(yangjinlong2@genomics.cn)](mailto:(yangjinlong2@genomics.cn))

**Supplement**

**Supporting Information Legends**

1. Supplement.doc contains 3 sections. The 1st section is the R code used in association mining. The 2nd section is the description of all the data tables used in this study. The tables present separately the analyzed data, the results and the code names. The 3rd section is an interpretation of the previous article, titled “To investigation on anemia among the children of Dai in different regions of Yunnan”. The interpretation was cited several times in our article.
2. S1_Table.xls contains multiple tables, including the tables for the analyzed data and the results. A detailed description of each table can be found in Supplement.doc.
3. S2_Table.xls is a table that presents the code names. For convenience, each mutation was assigned a code name. The association rules are described with the code name of the mutation.

**The 1st section**

**R code used in association mining**

library(arules)

DataTable1 # All data in a region, all sequence types are presented with code names.

Rules.All <- apriori(DataTable1, parameter = list(minlen=2, supp=0.01, conf=0.1), control = list(verbose=F)) # Look for assciation rules

Rules.All.Sorted <- sort(Rules.All, by = "lift") # Sort the rules by "lift"

Rules.All.Subset <- is.subset(Rules.All.Sorted, Rules.All.Sorted)

Rules.All.Subset[lower.tri(Rules.All.Subset, diag=T)] <- FALSE

redundant <- colSums(Rules.All.Subset, na.rm=T) >= 1

Rules.All.Pruned <- Rules.All.Sorted[!redundant] # Remove redundant rules

Rules.All.Pruned.Table <- as(Rules.All.Pruned, "data.frame")

**The 2nd section**

Table list

2.1 Code name table

S2_Table.xls is a collection of code name tables. Each sequence type (norm or pathogenic mutation, copy number variation) found by genetic test was allocated a code name much shorter than the annotation. Thus the trouble due to the long text of annotation was avoided when plotting or writing table.

2.2 Data table

S1_Table.xls, contains the analyzed data, and the tables for the analyzed data and results.

Table “DataForAna” is the data table analyzed in our research. The data was previously published in article “Next-generation sequencing improves thalassemia carrier screening among premarital adults in a high prevalence population: the Dai nationality, China”. Each manual inputted iterm in raw data was assigned a standardized description. No sequence or copy number variation was typed by us here. The description ensures a one to one correspondence between sequence type and annotation text. Meanwhile all escape characters were removed.

Table "AssociationRulesBN" and "AssociationRulesDH" are the lists of the association rules. The former is for Xishuangbanna, and the later for Dehong. The properties "support", "confidence" and "lift" are interpreted in R package "aRules".

Table "FTestAssociationRulesBN" and "FTestAssociationRulesDH" are the results of association rules' significance test. The properties in the tables accord to the output of R function "fisher.test".

Table "ComparisonBN" and "ComparisonDH" are generated when discussing whether the apriori algorithm found rules can be replaced by simpler rules. "Rule" is the apriori algorithm found rule; "SimplerRule" is the simpler rule that could replace apriori algorithm found rule; "N1", "N2", "N3", "N4" are all integers (see Method); The rest are the result of fisher test(see Method).

Table "RuleBNInDH" is for discussing whether the apriori algorithm found rule is significant in Dehong when it is significant in Xishuangbanna. Property "{lhs&rhs}" is the sample number in Dehong according to both the lhs and rhs of the given rule; "{lhs}" is the sample number according to the lhs; "{rhs}" is the sample number according to the lrhs, "{all}" is the total sample number in Dehong. The rest properties are for the result of fisher test.

Table "CompareTypeFrequency01" is for sequence type (or copy number variation) comparison between Xishuangbanna and Dehong. It lists the number and frequency of each type, and the result of the fisher test.

Table "CompareTypeFrequency02" is for genotype comparison between Xishuangbanna and Dehong. The genotype is described with the combination of sequence types, see property "Genotype_Info". Each genotype's count and frequency are also listed, and the result of fisher test are recorded.

Table “PermutationTestBN” and “PermutationTestDH” are the result of permutation test. Each row is for a rule. Each column is the proportion Fisher test p values less than a given value. For example, column “p.01” is the proportion of Fisher test p values less than 0.01. The detail of permutation test is described in Method.
